# Response consistency and response time across later life stages: evidence from a factorial survey on housing decisions

**DOI:** 10.1093/geroni/igag097

**Published:** 2026-08-28

**Authors:** Manuela Schulz, Christiane Gross, Andrea Teti

**Affiliations:** Institute of Gerontology, University of Vechta, Vechta, Germany; Institute of Political Science and Sociology, University of Würzburg, Würzburg, Germany; Institute of Gerontology, University of Vechta, Vechta, Germany

**Keywords:** Vignette study, Response behavior, Age-related heterogeneity, Compensatory strategies, Data quality

## Abstract

**Background and Objectives:**

Factorial surveys are increasingly used in gerontological research to study complex decision-making in later life. While prior work has demonstrated their feasibility among older adults, less is known about how response behavior varies across later-life stages and how response time relates to response consistency, particularly among the oldest old.

**Research Design and Methods:**

Data are drawn from a factorial survey on housing decisions conducted with a convenience sample of adults aged 50 to 99 years in Germany (*N* = 266). Response inconsistency was operationalised as a respondent-level measure based on residual variation in vignette evaluations. Regression and interaction analyses were used to examine age-related patterns and the relationship between response time and response inconsistency.

**Results:**

Response time increased with age. Response inconsistency did not vary across most age groups but was higher among the oldest respondents. No statistically robust association between response time and response inconsistency was observed, and interaction effects were not significant. Exploratory analyses indicated that age-related differences in inconsistency were detectable only within a limited range of response times.

**Discussion and Implications:**

The findings emphasise the importance of differentiating between stages of later life when assessing survey response behavior. They suggest that response pace and response consistency capture distinct dimensions that should not be treated as interchangeable indicators of data quality. By focusing on age-specific patterns, the study contributes to a more differentiated interpretation of response behavior in gerontological survey research.

Innovation and Translational Significance: Factorial surveys are an increasingly used method to study decision-making in later life. This study examines how response time and response consistency relate across different stages of later life using a vignette-based survey. The findings show that slower responding at higher ages is not systematically associated with less consistent evaluations. By distinguishing between response pace and response consistency, the study contributes to a more differentiated understanding of response behavior in cognitively demanding survey designs. This supports the use of complex survey methods in heterogeneous older populations and helps ensure that findings are interpreted appropriately in research, policy, and practice.

## Background and objectives

Innovation in gerontological research is often associated with the development of new interventions, technologies, or models of care. However, innovation also occurs more incrementally through the refinement of research methods and the expansion of populations included in empirical inquiry. One area in which such developments are particularly visible is survey research, where efforts to better capture the perspectives and decision-making processes of older adults have led to the increasing use of more complex and context-sensitive designs. Among these, factorial surveys have emerged as a promising approach for studying multidimensional decision-making in later life. By presenting respondents with systematically varied scenarios, factorial surveys allow researchers to approximate real-life judgments more closely than traditional survey formats (Teti et al., 2016).

As factorial surveys have been increasingly applied to aging populations, methodological discussions have begun to address key challenges associated with their use. In particular, prior research has focused on respondent burden, dropout, and the consistency of vignette evaluations as indicators of participants’ ability to engage meaningfully with cognitively demanding tasks (Shamon et al., 2022). Previous methodological research on factorial surveys has frequently examined feasibility, presentation formats, response time, and response consistency (Sauer et al., 2011; Shamon et al., 2022; Treischl & Wolbring, 2022). Comparatively less attention has been paid to how age-related differences in these indicators are interpreted after data collection. In particular, higher age and longer response times are often discussed in relation to task difficulty and potential data-quality concerns (Isaksson et al., 2007; Kutschar et al., 2019; Sauer et al., 2011; Watson & Nesa, 2025). However, this relationship remains conceptually and empirically ambiguous. Gerontological research consistently emphasizes that later life is characterized by substantial heterogeneity rather than uniform decline (Baltes & Smith, 2003). Differences in response pace may therefore reflect not only cognitive constraints but also alternative strategies of engagement. In this context, taking more time may enable respondents, particularly at very advanced ages, to engage more carefully with complex tasks, without necessarily resulting in less consistent evaluations.

### Factorial surveys as an emerging method in gerontological research

Factorial surveys represent an experimental approach embedded within survey research in which respondents evaluate hypothetical scenarios that vary systematically across multiple dimensions. Rather than assessing isolated attributes, respondents provide holistic evaluations of complex situations (Wallander, 2009). This design is particularly well-suited to research questions involving multidimensional tradeoffs, such as weighing housing accessibility against housing costs or care needs against personal autonomy. Compared with traditional survey formats, vignette-based designs allow researchers to disentangle the relative importance of different attributes while providing respondents with a concrete and standardized evaluative context. The systematic variation and randomization of vignette dimensions further enable the estimation of causal effects of individual factors within population samples, including older adults (Stansbury & Ludwick, 2009; Van Der Pas et al., 2005; Van Loon & Decancq, 2022). Beyond their analytical advantages, factorial surveys offer several features that are particularly relevant for gerontological research. First, they enable the elicitation of older adults’ own preferences and evaluative criteria, thereby supporting more person-centred and preference-based evidence for policy and practice (Van Loon & Decancq, 2022). Second, they allow the study of sensitive or rare situations, such as end-of-life decisions, relocation, or family support, without exposing participants to real-world risks, enhancing both ethical and practical feasibility (Teti et al., 2016; Van Der Pas et al., 2005; Van Loon & Decancq, 2022). Taken together, these features make factorial surveys a particularly valuable tool in gerontology. They allow researchers to approximate complex real-life decisions, quantify how different factors shape older adults’ judgments, and do so in a way that is both experimentally rigorous and feasible for use with older populations.

For example, Van Loon and Decancq (2022) presented older respondents with hypothetical persons whose situations were described using varying combinations of six dimensions: health, income, social relations, leisure, engaging activities, and religion. Respondents rated each person’s overall well-being, allowing the researchers to estimate the extent to which each dimension contributed to the overall judgment of well-being. This illustrates how factorial surveys combine several independently varying characteristics within a single vignette rather than asking respondents to evaluate each dimension in isolation.

Early applications of factorial surveys in gerontological research appear relatively recent. Existing studies typically treat the method as already established and apply it to older populations rather than documenting its initial introduction into the field. To our knowledge, one of the earliest studies explicitly involving respondents aged 60 and older examined health information–seeking behavior using a factorial survey design (Stansbury & Ludwick, 2009). Other vignette-based studies addressed gerontological topics but often relied on younger samples (e.g., Killick & Taylor, 2012). Consistent with this pattern, a methodological review of factorial surveys in sociology noted that the approach had been applied predominantly to younger populations, such as student samples and professional groups (Wallander, 2009).

Since around 2010, factorial survey and vignette-based designs have become more widely used in gerontological research. Methodological reviews document a broader expansion of factorial survey experiments across the social sciences, including applications in health and aging research (Dülmer, 2016; Treischl & Wolbring, 2022). More recent studies illustrate the growing substantive use of these designs. For example, a large-scale factorial survey among older adults in Flanders quantified the relative importance of different well-being dimensions, including health, income, and social relations (Van Loon & Decancq, 2022). Similarly, vignette-based approaches have been used to study sensitive topics such as the recruitment of older African American adults into research, demonstrating their suitability for contexts involving trust and ethical considerations (Gamboa et al., 2021).

Across this body of work, factorial and vignette-based studies generally do not differentiate explicitly between stages of later life, such as “younger-old” and “oldest-old” populations. Instead, age is typically treated as a continuous variable, often alongside indicators of cognitive status or educational attainment.

### Response time and response consistency: a missing link

Factorial surveys are cognitively demanding, as respondents must repeatedly process and integrate multiple pieces of information across several scenarios. This has raised questions about their suitability for older populations. Early methodological research, therefore, focused on whether older adults are able to complete such tasks and provide consistent evaluations. Overall, these studies suggest that factorial surveys can be applied successfully in older populations, provided that design complexity is carefully managed and appropriate support is available (Sauer et al., 2011; Teti et al., 2016; Van Loon & Decancq, 2022). Limitations are most evident in subgroups with lower levels of education or cognitive impairment, where simplified designs or alternative approaches may be required (Sauer et al., 2011).

Within this literature, response consistency and response time have emerged as key indicators for assessing response behavior. Response consistency has been widely examined, whereas response time has received more differentiated attention in recent years. Across survey research more broadly, longer response times are often interpreted as reflecting increased cognitive effort or task difficulty. At the same time, this interpretation is recognized as incomplete, as response times may also be influenced by distraction, multitasking, or individual response styles (Hu et al., 2022; Junghaenel et al., 2022). Empirical studies indicate that response time is related to common indicators of data quality, such as instruction compliance or response coherence, but that these relationships are moderate and heterogeneous (Cohen et al., 2021). In many survey contexts, extremely short response times are consistently associated with lower response quality, supporting their use as indicators of insufficient engagement (Atzori et al., 2023; Bowling et al., 2023; Wood et al., 2017). Beyond this, however, the relationship between response time, cognitive burden, and response quality is more nuanced. Some approaches distinguish between systematic adjustments of response time to task demands, potentially reflecting adaptive engagement, and unsystematic fluctuations that may indicate lower data quality (Bago d’Uva et al., 2009; Grol-Prokopczyk et al., 2015; Lenzner et al., 2010). Evidence from factorial survey research supports this distinction. For example, older respondents have been found to exhibit longer raw response times without corresponding increases in cognitively controlled latency measures, suggesting that slower responding does not necessarily reflect greater cognitive burden (Sauer et al., 2011).

This distinction is particularly relevant in the context of aging. It is well established that response times tend to increase with age, reflecting changes in processing speed as well as differences in task engagement. At the same time, research on cognitive aging emphasizes that slower responding may also reflect adaptive strategies. Older adults often adopt more cautious decision-making strategies, allocating additional time to process information and maintain accuracy in cognitively demanding tasks (Baltes & Smith, 2003; Vallesi et al., 2021). Experimental and modeling studies suggest that older individuals may set higher response thresholds and accumulate more information before responding, which can contribute to slower but not necessarily less consistent performance (Dubravac & Meier, 2023; Pournaghdali & Eich, 2025). From this perspective, longer response times should not be interpreted in a unidimensional way. While they may indicate increased task difficulty in some cases, they may also reflect more deliberate engagement with complex tasks. Importantly, longer response times do not necessarily coincide with greater response inconsistency (Sauer et al., 2011).

Finally, existing factorial survey research has rarely differentiated explicitly between stages of later life, such as younger-old and oldest-old populations. This distinction may be relevant, as individuals in advanced stages of later life differ not only in levels of functional limitation but also in available resources and compensatory strategies (Allen & Windsor, 2019; Baltes & Smith, 2003). A more differentiated perspective on age may therefore be important for understanding variation in response behavior within older populations.

Building on these considerations, the present study examines how age, response time, and response inconsistency are related in a factorial survey context. The existing literature suggests that response consistency and response time capture distinct dimensions of response behavior, while their interrelationship, particularly across different stages of later life, remains insufficiently understood.

### Hypotheses

Building on the considerations above, this study examines three hypotheses regarding response inconsistency in vignette-based surveys among older adults.

Hypothesis 1 proposes that response inconsistency varies across later-life stages rather than increasing uniformly with age. Specifically, higher levels of response inconsistency are expected to be more pronounced among very old respondents, whereas younger-old age groups are not assumed to differ systematically. This reflects the notion that older adults should not be treated as a homogeneous group and that age-related variation may be concentrated in advanced stages of later life.

Hypothesis 2 focuses on age-related differences in response pace. Based on research on cognitive aging and adaptive strategies, older respondents are expected to require or take more time when evaluating complex scenarios. Accordingly, response time is expected to increase with age.

Hypothesis 3 proposes that the relationship between response time and response inconsistency differs across age groups. In particular, shorter response times are expected to be more strongly associated with lower response consistency among the oldest respondents. This expectation reflects the idea that taking more time may facilitate more structured evaluations in cognitively demanding tasks (see Figure 1).

**Figure 1:**
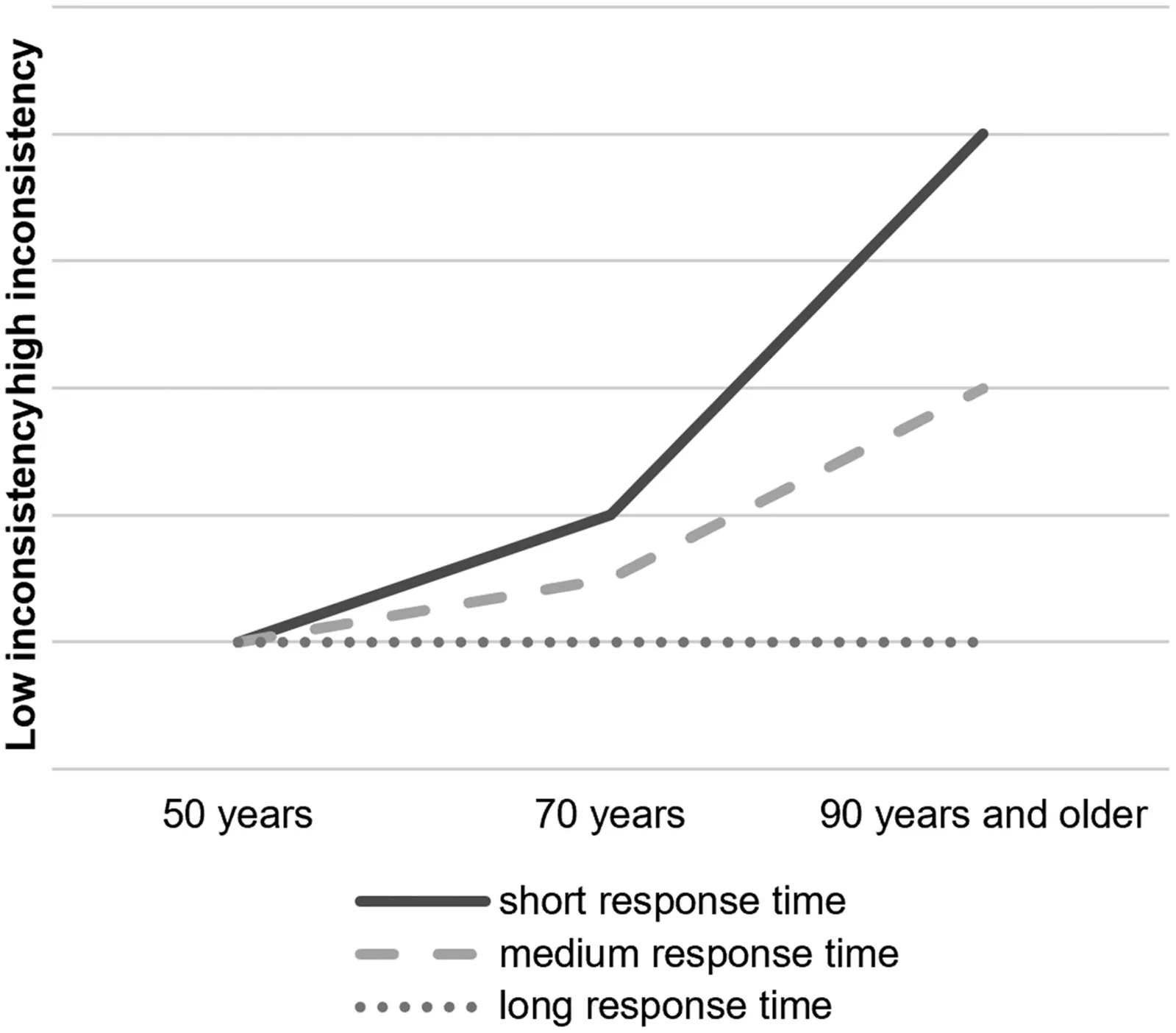
Conceptual illustration of the hypothesized relationship between inconsistency, age, and response time.

## Research design and methods

### Design and sample characteristics

The study draws on data from a vignette-based factorial survey on housing decisions conducted among older adults in Germany (anonymised). Participants were recruited between 2018 and 2023 through the interviewers’ extended personal networks, including local community members and informal senior networks. This purposive recruitment strategy does not allow for statistical generalization but enables the inclusion of older adults living in a wide range of private housing situations. However, this strategy likely favored socially connected adults, while individuals with severe health, cognitive, or social limitations may have been underrepresented. The sample should therefore not be considered representative of older adults living in private households in the region. Eligibility criteria required respondents to be aged 50 years or older and to reside in private housing at the time of the interview.

A total of 332 individuals participated in the study. For the present analyses, 266 respondents were included based on the availability of complete sociodemographic information, information on current living conditions, and valid evaluations for at least 9 out of 10 vignettes. Similar vignette-based studies have relied on comparable or smaller samples when examining response patterns at the respondent level (Sauer et al., 2011; Teti et al., 2016). Sample characteristics are presented in Table 1.

**Table 1:** Sample characteristics.

Variables	*N*	**%**	Mean	*SD*	Min	Max
**Age (years)**						
50–59	83	31				
60–69	107	40				
70–79	42	16				
80–99	34	13				
**Educational level (CASMIN)**						
No school qualification or low educational level	32	12				
Intermediate educational level	163	61				
High educational level	71	27				
**Equivalent income (monthly)**						
<€1,250	83	32				
€1,250–3,000	161	59				
>€3,000	22	8				
**In paid employment**						
Yes	125	47				
No	141	53				
**Ownership**						
Rent or other	69	26				
Owner	197	74				
**Gender**						
Female	149	56				
Male	117	44				
**Intention to move**						
Yes	73	27				
No	193	73				
**Size of household**						
Single household	70	26				
Two-person household	154	58				
More than a two-person household	42	16				
Age in years			65.03	10.21	50	99
Duration of residence in years			24.17	16.98	1	90
Size of the apartment in sqm/person			70.65	32.50	16.67	200
Net household equivalent income			1,770.30	1,053.81	100	8,000
Rent of apartment in €/person^a^			570.62	298.85	36.67	2,500
Response time (in min)			12.61	7.32	2	50

*Note*. CASMIN = comparative analyses of social mobility in industrial nations. *N *= 266.

a Or the estimated rent if a person owns property.

The survey was administered in a face-to-face interview setting using pen-and-paper questionnaires. Interviewers were present throughout the vignette task and assisted when necessary, for example, in cases of visual impairment or when questions arose about task instructions. At the same time, interviewers did not guide or influence respondents’ evaluations of the vignette content. This interview context was chosen to facilitate participation across a broad range of older age groups while maintaining a standardized presentation of the vignette material.

The vignette design followed a factorial survey approach in which housing situations were described by a fixed set of experimentally varied attributes. The complete factorial design comprised 128 unique vignettes, generated by combining seven housing-related dimensions, each with two levels (for more information see Supplementary Table 1). From this universe, a subset of 100 vignettes was randomly selected and divided into 10 sets of 10. The allocation of vignettes to sets, as well as the order of vignettes within each set, was randomized. Once assigned, the order within a set was held constant and identical for all respondents evaluating that set. Each respondent evaluated a set of 10 vignettes and rated the likelihood of moving into the described housing options. Ratings were provided on a fully visible 10-point scale ranging from 1 “very unlikely” to 10 “very likely,” with verbal anchors displayed at both endpoints. The vignettes were presented in printed form, and respondents completed the task individually in the presence of a trained interviewer. The selection of dimensions and levels was informed by prior research on residential relocation in later life and by earlier applications of similar vignette materials in related research contexts (Teti et al., 2016). Before the main field phase, the vignette material underwent iterative testing and refinement to ensure clarity, plausibility, and comprehensibility. An example vignette is provided in Figure 2.

**Figure 2:**
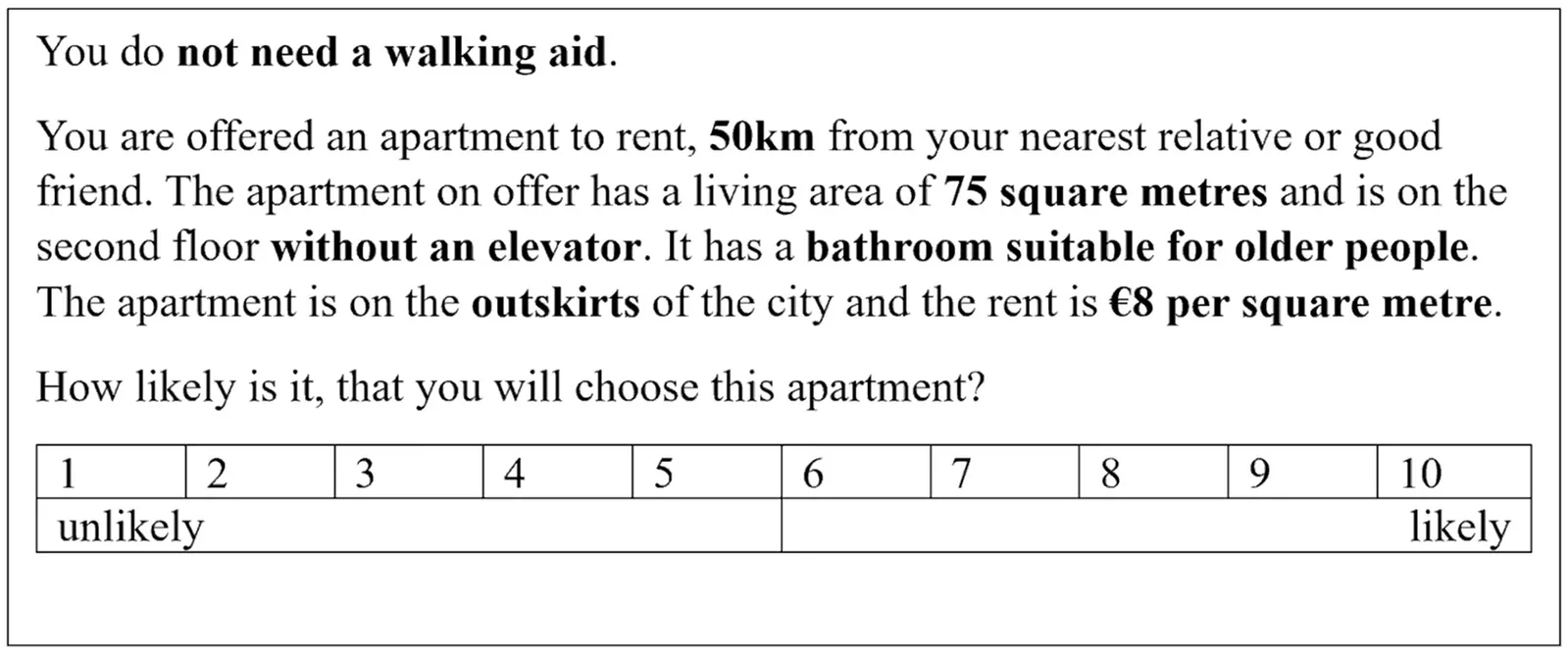
Example vignette.

### Measures

#### Response inconsistency

The primary outcome of this study is a respondent-level indicator of response inconsistency, intended to capture the extent to which participants’ vignette evaluations systematically reflect the experimentally varied housing attributes. For each respondent, a linear regression model was estimated with the 10 vignette ratings as the dependent variable and the seven vignette dimensions as predictors. The absolute residuals from these regressions represent deviations from a consistent, model-based evaluation pattern. We calculated the mean absolute residual per respondent as an index of response inconsistency, with higher values indicating greater variability in evaluations unexplained by the vignette attributes alone. This approach follows established applications in vignette research that derive respondent-level inconsistency measures from residual variation in individual vignette evaluations (Sauer et al., 2011), while using mean absolute residuals to reduce sensitivity to outliers on bounded rating scales.

It is important to note that the inconsistency indicator is based on a limited number of vignette evaluations per respondent. Each respondent evaluated 10 vignettes, while the individual regression model includes seven vignette dimensions and an intercept. Consequently, the residual variation underlying the inconsistency index is estimated with a small number of degrees of freedom. Individual inconsistency scores, therefore, contain measurement noise and should not be interpreted as precise individual traits. Rather, the indicator is intended to capture response patterns at the respondent level and is suitable for analyzing group-level associations, which is the focus of the present study.

#### Response time

Response time was measured as the total time respondents spent completing the vignette task. Given the face-to-face interview setting and the use of printed vignettes, measured response times may include components unrelated to cognitive processing, such as differences in reading speed, brief interactions with the interviewer, or support provided during the task. Accordingly, variation in response time reflects the overall interactional pace of the interview situation rather than isolated cognitive processing speed.

#### Control variables

Control variables were selected based on plausible mechanisms linking age to response behavior. Education, income, and employment status may reflect socioeconomic and cognitive resources that support engagement with complex survey tasks. Household size may capture differences in social context and support, while gender may be associated with age-related differences in socioeconomic and housing circumstances (Sauer et al., 2011; Teti et al., 2016). These variables were therefore included to account for potential confounding of the association between age and response inconsistency.

Age was measured in years. Educational attainment was categorized into three levels: (1) no school-leaving qualification or low educational level (primary education), (2) intermediate educational level (lower and upper secondary education), and (3) high educational level (tertiary education, including bachelor’s, master’s, and doctoral degrees).

Household economic resources were captured using net equivalent household income, calculated as total household income divided by the number of household members. Employment status was recorded as “in paid employment” versus “not in paid employment,” with retirement included in the latter category.

Several variables related to respondents’ housing situations were also included. Duration of residence was reported in years. Household size reflects the number of individuals living in the household, including the respondent. Dwelling size was measured as square meters per person, and monthly rent was recorded in Euros.

Finally, respondents’ intention to move was included as an indicator of the vignette topic’s relevance. Responses were dichotomized to distinguish between respondents who reported at least some intention to move and those who reported no intention to move. All control variables were included to reduce potential confounding and are not associated with separate hypotheses.

### Analysis

All analyses were conducted at the respondent level. The primary outcome variable, response inconsistency, was derived as a single summary indicator per respondent based on their vignette evaluations. Consequently, each respondent contributes one observation to the analytical models. This analytical strategy focuses on associations between respondent characteristics and response patterns, rather than on modeling vignette-level responses. Age was grouped into decade-based categories to reflect heterogeneity across later-life stages rather than assuming a linear age effect. Response time was categorized based on the turning points (short response time < 15 min, *N* = 200; medium response time = 15.0–29.9 min, *N* = 56; long response time ≥ 30min, *N* = 10).

To examine associations between response inconsistency, age, and response time, we estimated ordinary least squares regression models. In a first step, age and the set of control variables were included to assess baseline associations with response inconsistency. In a second step, response time was added to examine its relationship with response inconsistency net of age and other covariates. In a third step, an interaction term between age and response time was included to assess whether the association between response time and response inconsistency varies across age.

Interaction effects were examined using standard product terms. To aid interpretation, conditional associations between response time and response inconsistency across the age range were probed using Johnson–Neyman techniques. These analyses are intended to illustrate age-related heterogeneity in associations rather than to identify sharp thresholds or discontinuities. Before analysis, all continuous variables were inspected for outliers and plausibility. All analyses were conducted using R (version 4.2.2).

## Results

### Age-related patterns in inconsistency and response time

As a first step, we examined age-related patterns in response inconsistency and response time. Figure 3 displays the bivariate associations between age, response time, and the residual-based inconsistency index.

**Figure 3:**
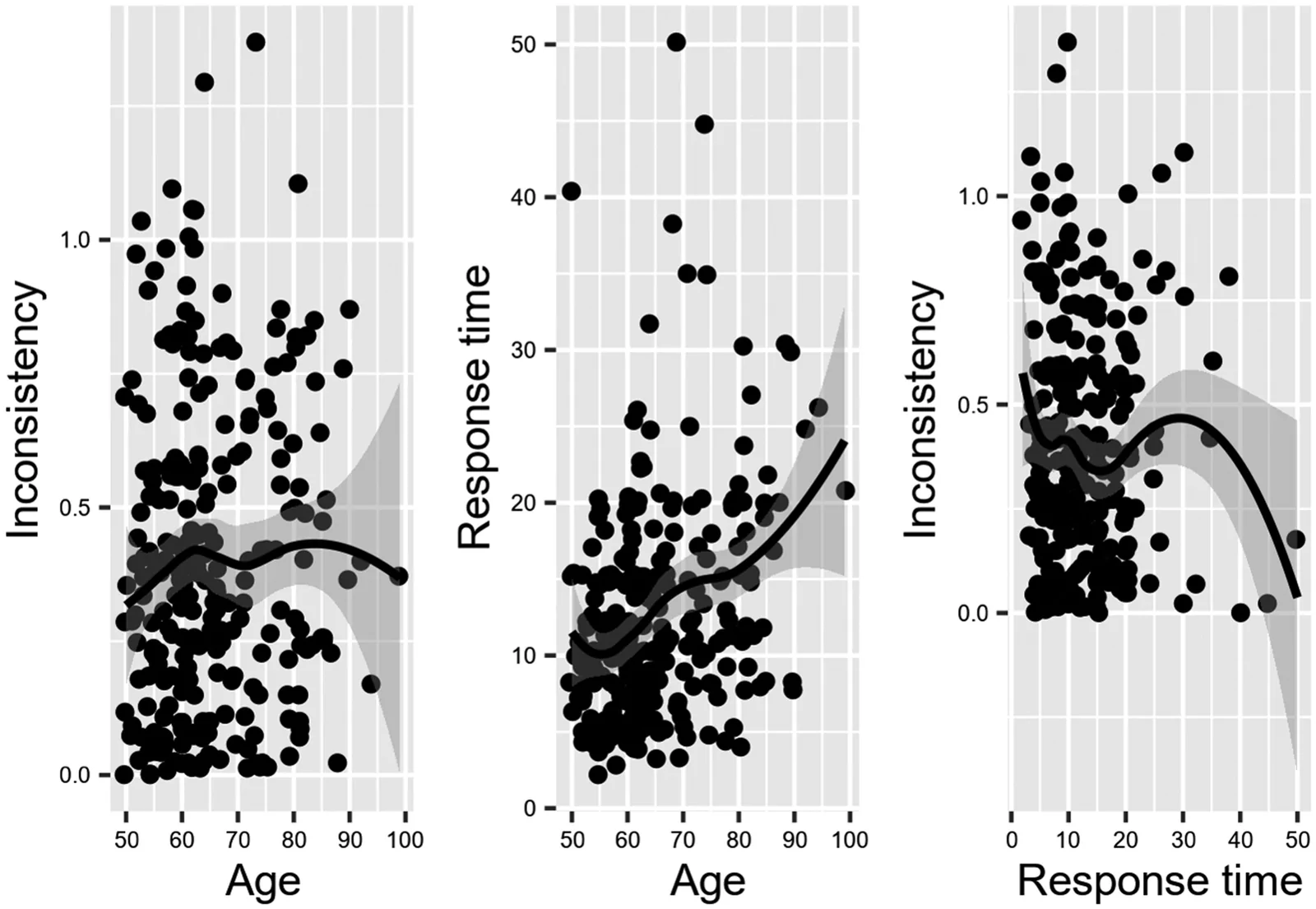
Associations between response time, inconsistency, and age.

**Figure 4:**
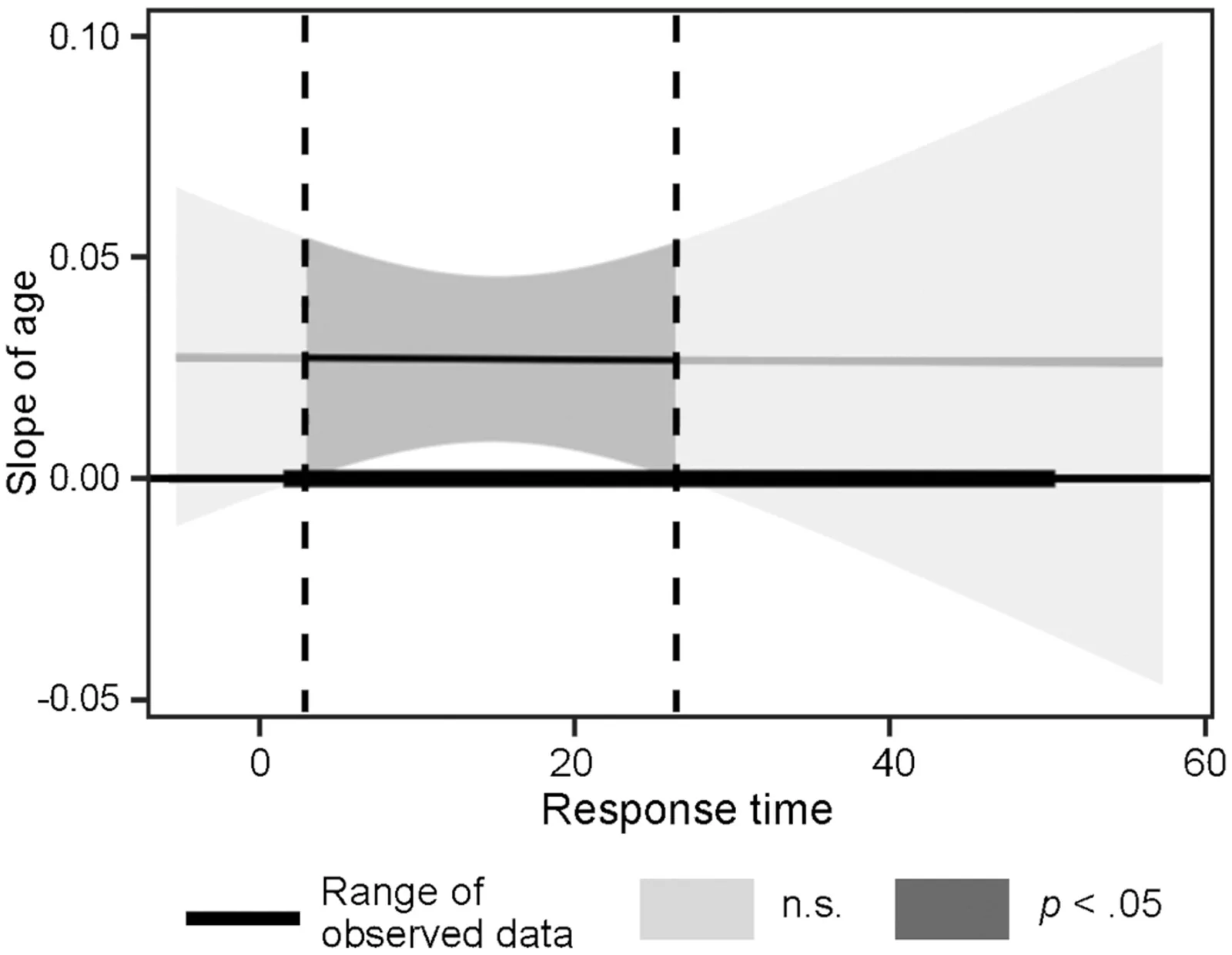
Johnson–Neyman output from the interaction of age and inconsistency and response time as moderator variable. n.s. = not significant.

At the descriptive level, age was positively associated with response time (*r* = 0.31, *p* < .001), indicating that older participants tended to take more time to complete the vignette task. In contrast, no clear linear association was observed between age and response inconsistency.

Multivariate regression models focusing on response inconsistency revealed no systematic differences between participants aged 60–69 or 70–79 and the reference group (50–59). Only respondents in the oldest age group (80–99) showed higher average residual variation across vignette ratings (*β* = .23, *p* < .05).

Taken together, these results indicate that age-related differences in response behavior are domain-specific. While response time increases gradually with age, differences in response inconsistency are confined to the highest age group rather than reflecting a continuous age gradient.

### Response time as moderator of age differences in inconsistency

To examine whether the association between age and response inconsistency varies by response time, we estimated regression models including interaction terms between age groups and response-time categories.

None of the interaction terms reached conventional levels of statistical significance. Accordingly, the multivariate models do not indicate a robust moderating effect of response time on age-related differences in response inconsistency. For the full results of the regression models, see Supplementary Table 2.

Additional Johnson–Neyman analyses were conducted to explore conditional associations across the continuous range of response time (see Figure 4). These analyses showed that the effect of age on response inconsistency was positive and statistically distinguishable from zero only within a limited range of response times (2.3–25.4 min). Outside this interval, the association was not statistically significant. Given that only 10 respondents had response times of 30 min or longer (one respondent aged 50–59 years, three aged 60–69 years, three aged 70–79 years, and three aged 80–99 years), these findings are reported as descriptive patterns and are not interpreted as confirmatory evidence for Hypothesis 3.

## Discussion and implications

### Summary and interpretation of findings

This study examined age-related patterns in response behavior within a factorial survey context. Across the analyses, three central findings emerge. First, age-related differences in response inconsistency were not gradual or linear. For respondents aged 79 or younger, response inconsistency did not differ systematically from that of younger participants. Only in the highest age group did the residual-based inconsistency index indicate higher unexplained variation in vignette evaluations.

Second, response time increased monotonically with age. Older respondents tended to take more time to complete the vignette task. Importantly, this pattern was not accompanied by a general increase in response inconsistency, underscoring the conceptual distinction between response pace and response consistency.

Third, contrary to expectations, response time did not robustly moderate age differences in response inconsistency. While exploratory analyses suggested that age-related differences in inconsistency were observable only within a restricted range of response times, these patterns were not supported by statistically significant interaction effects and should therefore be interpreted descriptively.

Previous research has shown that factorial surveys can be applied successfully in later life and that response time tends to increase with age, whereas evidence on response consistency has been inconsistent across studies. The present results are broadly in line with these findings. They further support the view that factorial surveys are a feasible method for both older and very old populations, and confirm that response time increases with age, without necessarily being associated with greater response inconsistency.

Regarding the relationship between response time and response inconsistency, the analyses do not provide robust evidence of a moderating effect. While regression models did not yield statistically significant interaction terms, exploratory Johnson–Neyman analyses suggest that age-related differences in response inconsistency may emerge within specific ranges of response time. In particular, the association between age and inconsistency was detectable only within a mid-range interval of response times, whereas no such pattern was observed at shorter or longer durations. These findings should be interpreted with caution, given the limited number of observations at longer response times and the absence of statistically significant interaction effects. However, they indicate that the relationship between response pace and response consistency may not be uniform across the full range of response times. Rather than pointing to a general moderating effect, the results suggest that associations may be conditional and context-dependent. Importantly, the findings do not support the assumption that allocating more time systematically leads to more consistent responses in later life. At the same time, they do not contradict the idea that response time may reflect adaptive engagement strategies. Instead, the results indicate that such strategies may not translate directly into measurable differences in response consistency, at least not in a uniform or linear way.

### Limitations

Several limitations that may affect the interpretability of the findings should be acknowledged. First, the study is based on a convenience sample, which precludes statistical generalization. The analyses are therefore intended to identify patterns in response behavior rather than to estimate population-level effects.

Second, the number of respondents in the highest age group was limited, particularly among those with very long response times. This constrains the statistical power to detect interaction effects and warrants caution when interpreting exploratory moderation patterns.

Third, response inconsistency was operationalized as a residual-based index derived from a limited number of vignette evaluations per respondent. While this approach captures systematic deviations in vignette attributes, it is inherently noisy and not well suited for individual-level assessment.

Finally, data were collected cross-sectionally over an extended period. Although the vignette instrument and administration procedures remained constant, temporal and cohort-related influences cannot be disentangled and may have contributed to unobserved heterogeneity in response patterns.

### Implications for methodological development in gerontological survey research

The contribution of this study is modest but relevant. By examining the relationship between response time and response inconsistency in a sample covering a wide age range, including very old respondents, the analyses provide additional empirical support for distinguishing between response pace and response consistency in later life. The results do not provide robust evidence that allocating more time systematically supports more consistent evaluations in later life. This does not invalidate the notion of adaptive response strategies, but suggests that their effects may not be directly observable in response consistency measures alone.

At the same time, the findings point to an area in which further methodological development may be useful. While gerontological research has made substantial progress in applying factorial surveys to older populations and in assessing response consistency, the relationship between response time and response consistency has received comparatively limited attention.

From a design perspective, the results suggest that allowing sufficient time for engagement with complex tasks may be beneficial, particularly in heterogeneous populations. More broadly, the findings contribute to ongoing efforts to refine the conceptualization of data quality in survey research with older populations. Rather than focusing primarily on whether older adults can engage with complex survey designs, future research may benefit from examining how different dimensions of response behavior relate to one another and how they can be interpreted in a more differentiated manner.

## Conclusion

This study contributes to methodological research on factorial surveys by providing a more differentiated perspective on response behavior in later life. While response pace increased with age, response inconsistency did not show a general age gradient and was confined to a small subset of the oldest respondents.

These findings underscore the importance of analytically separating response pace from response consistency and avoiding the use of observable characteristics, such as age or response time, as indirect indicators of data quality. Instead, response behavior should be interpreted in a way that accounts for the distinct dimensions it comprises.

## Supplementary Material

igag097_Supplementary_Data

## Data Availability

The syntax supporting the conclusions of this article is available in the figshare repository https://figshare.com/s/dd508653087bd5506236. The dataset is published at GESIS repository. Teti, A., & Schulz, M. (2026). HOME II *(Version 1.0.0) [Data set]. GESIS, Cologne.*  https://doi.org/10.7802/2659. This research was not preregistered.
