# Supplementary material for: Response consistency and response time across later life stages: evidence from a factorial survey on housing decisions
Source: Innov Aging. 2026 Aug 28;10(11):igag097. doi: 10.1093/geroni/igag097 (PMC13719224; doi:10.1093/geroni/igag097)
Supplement: Supplementary file 1 [file igag097_supplementary_data.pdf]

Innovation in Aging **Supplementary Material: Schulz, Gross, & Teti. Response consistency and response time across later life stages: Evidence from a factorial survey on housing decisions.**

Supplementary Table 1: Vignette dimensions

Dimension	Category 1	Category 2
Mobility	Do need a walking aid.	Do not need a walking aid.
Social network	5km from your nearest relative or good friend	50km from your nearest relative or good friend
Apartment size	75 sqm	45 sqm
Household amenities 1	with an elevator	without an elevator
Household amenities 2	bathroom suitable for older people	bathtub
Living area	city centre	outskirts
Rent	€8 /sqm	€12 /sqm

Supplementary Table 2: Linear regression models with inconsistency as outcome

	Model 1			Model 2			Model 3		
**Variables**	**β**	**SE**	**p**	**β**	**SE**	**p**	**β**	**SE**	**p**
Age (Ref.: 50-59)									
60–69	0.13	0.08	0.080	0.13	0.08	0.071	0.11	0.08	0.154
70–79	0.14	0.09	0.113	0.15	0.09	0.106	0.15	0.10	0.114
80–99	0.23	0.09	0.010	0.24	0.09	0.009	0.23	0.10	0.023
Gender (Ref.: male)	0.03	0.06	0.577	0.03	0.06	0.596	0.02	0.07	0.767
Education (Ref.: low)									
medium	0.11	0.10	0.272	0.11	0.10	0.269	0.15	0.11	0.152
high	0.16	0.11	0.132	0.16	0.11	0.138	0.17	0.11	0.112
Income	0.01	0.08	0.920	0.01	0.08	0.938	0.03	0.08	0.728
Employed	0.02	0.09	0.724	0.02	0.09	0.729	0.02	0.09	0.759
Relevance (Ref.: not present)	-0.01	0.06	0.825	-0.02	0.06	0.809	-0.01	0.07	0.883
Duration of residence in years	-0.18	0.07	0.013	-0.18	0.07	0.012	-0.17	0.07	0.017
Household size	0.04	0.08	0.650	0.04	0.08	0.675	0.05	0.08	0.611
Size of apartment/person in sqm	-0.01	0.10	0.910	-0.02	0.10	0.868	0.01	0.10	0.892
Rent/person in Euro	-0.04	0.10	0.667	-0.04	0.10	0.723	-0.07	0.10	0.510
Response time (RT)									
short RT (<15min)				0.01	0.14	0.988	0.16	0.18	0.390
medium RT (15–29.9min)				-0.03	0.14	0.801	0.16	0.18	0.397
Age x Response time (RT)									
60–69: short RT							-0.19	0.25	0.440
70–79: short RT							-0.13	0.19	0.496
80–99: short RT							-0.28	0.18	0.117
60–69: medium RT							-0.25	0.25	0.322
70–79: medium RT							-0.25	0.19	0.199
80–99: medium RT							-0.32	0.17	0.068
Constant	0.00	0.06	1.000	0.00	0.06	1.000	0.01	0.07	0.933
Observations		266			266			266	
R^2		0.05			0.05			0.07	
Adjusted R^2		0.003			-0.004			-0.01	
Residual SE		1.00 (df = 252)			1.00 (df = 250)			1.00 (df = 244)	
F Statistic					1.06 (df = 13; 252)			0.93 (df = 15; 250)	

Note. *N* = 266.
